# Analysis of the Impact of Changes in Thermomechanical Properties of Annealed Semi-Crystalline Plastics on the Surface Condition after the Machining Process

**DOI:** 10.3390/ma16134816

**Published:** 2023-07-04

**Authors:** Adam Gnatowski, Rafal Golebski, Piotr Sikora, Jana Petru, Jiri Hajnys

**Affiliations:** 1 Department of Technology and Automation, Faculty of Mechanical Engineering and Computer Science, Czestochowa University of Technology, 42-201 Czestochowa, Poland; rafal.golebski@pcz.pl; 2 Production Services on Automatic Lathes, APJ Sikora, 42-200 Czestochowa, Poland; piotr@apjsikora.pl; 3 Department of Machining, Assembly and Engineering Metrology, Faculty of Mechanical Engineering, VSB—Technical University of Ostrava, 708 00 Ostrava, Czech Republic; jana.petru@vsb.cz (J.P.); jiri.hajnys@vsb.cz (J.H.)

**Keywords:** thermomechanical properties, melting, annealed, polymeric materials, machinability

## Abstract

In this paper, the authors present a comparative analysis of the thermomechanical properties of plastics intended for machining before and after the annealing process. The research included the dynamic properties, thermal analysis and a study of the surface after machining. The dynamic properties were tested using the DMTA method. The characteristics of changes in the value of the storage modulus E’ and the tangent of the mechanical loss angle tgδ depending on the temperature and vibration frequency were determined. The thermal properties were tested using the DSC method, and a comparative analysis of the roughness parameters of the tested materials obtained from the profilometer was carried out. The presented studies indicate the extent of the impact of the annealing process on the machinability of structural polymer materials, taking into account the analysis of changes in the thermomechanical properties of the tested materials.

## 1. Introduction

Polymer materials are characterized by different properties depending on the chemical composition and the method of their production. Many types of polymeric materials can successfully replace metal-based materials. Advanced methods for the production and processing of polymers mean that these materials are characterized by a very good ratio of mechanical properties in relation to their specific weight. Polymer plastics are an extremely versatile base for the production of all types of composites, often characterized by excellent mechanical properties. An example of this type of material is polymers reinforced with glass fibers, carbon fibers or metal particles. Plastics are often subjected to machining processing. This process is an alternative to obtaining the required shapes by means of plastic injection, pressing or casting. The greatest disadvantage of machining is the generation of a significant amount of waste when machining the material. However, the advanced technologies used in machining make it possible to obtain the highest accuracy and surface quality in the machined materials. Due to their structures and properties, some polymeric materials are quite easy to machine. Others, however, cause great difficulties in carrying out this type of technology—for example, polymers with mineral fillers, reinforced with glass fibers or characterized by high elasticity [1,2,3,4]. Thus, they do not belong to the group of easily machined materials. During the processing of polymeric materials, there are problems related to the plasticity, low Young’s modulus and low thermal conductivity of these materials. Excessive heating and surface pressure during machining can cause permanent deformation. The authors of [5] present a study of the mechanical properties and machinability of thermoplastic polymers. Through a dynamic mechanical analysis experiment, the modulus of elasticity and the effect of the temperature on the tested materials were analyzed. A high-speed milling experiment was conducted to evaluate the surface roughness, burrs and chip characteristics. The influence of the mechanical properties on the machinability and changes in the viscoelastic properties with increasing temperature were found. The modification of polymer-based materials in order to improve their mechanical properties often leads to an increase in the cutting force and, consequently, to an increase in the cutting temperature, which leads to the softening of the polymer material. Hence, it should be highlighted that the behavior of the polymer during machining is significantly different from that of metal alloys due to the dependence of the polymer on the temperature [6]. The problem of the influence of the machining parameters on the properties of a semi-crystalline polymer material has been considered by many authors [7,8,9]. The authors of [7] presented research aimed at assessing the machinability of typical thermoplastic and thermosetting polymers and understanding the impact of their viscosity on the surface integrity, chip formation and cutting force. Particular attention was paid to the interaction between the strain rate and temperature during processing. It was found that the viscous deformation of the polymer had a decisive influence on the quality of the machined surface. For example, to minimize surface roughness, machining conditions must be selected such that stock removal deformation is in the range beyond the viscoplastic and brittle fracture phases. The optimal processing conditions must be based on the polymer properties, such as the glass transition temperature, fracture toughness and the mobility of the polymer molecules.

In [8], both the mechanical properties and machinability of polyimide, a polymeric material with high heat resistance and good mechanical strength, are presented. Through a research experiment, the nanoindentation hardness and elastic modulus were analyzed. Test results indicated that polyimide had good mechanical properties and machinability. The authors of [9] studied the impact of the machining parameters on the technological parameters, surface roughness criteria and rate of material removal during polytetrafluoroethylene turning. Modeling of the output technological parameters was carried out in order to determine the most efficient process. The characteristics of cutting phenomena based on non-linear dynamics for the prediction of the transition from continuous chip formation to local shear formation in machining has been described in [10]. Experiments and a simplified model show that as the cutting speed increases, there is a shift from continuous to localized chip formation in the workpiece. With a further increase in the cutting speed, the average spacing between the shear bands increases monotonically, and the spacing becomes more regular and asymptotically approaches the limit value determined by the cutting conditions and the properties of the workpiece material. Consequently, it may be important in the case of machining semi-crystalline polymeric forms and their thermomechanical properties. Due to the ever-increasing requirements set by the processing industry and the implementation of modern technologies, it is necessary to optimize the properties of polymeric materials. One of the most interesting directions is the thermal treatment of polymeric materials in order to obtain optimal mechanical properties and improve the machinability after the annealing process. The research presented in [11] concerned changes in the thermomechanical properties and crystallization of poly(lactic acid) (PLA) composites. The method of crystallization of various composites was investigated using differential scanning calorimetry. The results obtained in the form of DSC thermograms of composites indicated an increase in the degree of crystallinity of materials along with the annealing process. After the annealing process at 100 °C, the authors of the research obtained an increase in the value of the heat distortion temperature (HDT) of the composites produced on the basis of PLA. Differential scanning calorimetry (DSC), dynamic mechanical analysis (DMA) and Fourier transform infrared (FTIR) techniques were used in [12] to explain the origins of beneficial changes in properties after the annealing of polymeric materials. The results suggest that the increase in crystallinity, glass transition temperature and degree of hydrogen bonding may be responsible for the noticeable increase in the HDT of polymer nanocomposites after the annealing process. The authors of [13] studied nanocomposites of polyamide 6 (PA6) with halloysite nanotubes. The samples were annealed for 3 h and then cooled down at a rate of 4 K/min. The authors’ research using differential scanning calorimetry confirmed that annealing increased the degree of crystallinity of the polyamide matrix. After thermal treatment, a nanocomposite with better mechanical properties was obtained. The authors of [14] presented a study of the effect of annealing on the polymorphic behavior and thermal properties of polyamide 6 as a function of the annealing time, using differential modulated scanning calorimetry (MDSC) and wide-angle X-ray diffraction, indicating significant differences in the thermal behavior and polymorphism of the tested materials before and after the annealing work. Morphological studies of PEEK [15] showed that annealing induces an increase in crystallinity and very significant differences in mechanical properties depending on the type of material reinforcement. The reduced values of the modulus of elasticity of the tested materials due to the addition of the modifier were also eliminated after the annealing process.

The present paper presents an analysis of the impact of polymer heat treatment on the milling process. The quantitative objective of the research was to determine the impact of annealing polymer materials on the quality parameters of the workpieces. In particular, attention was paid to the impact of thermal modification on the thermal properties, degree of crystallinity and thermomechanical properties of the materials. An analysis of the extent of the impact of the annealing process on the improvement in the machinability of structural polymer materials was carried out. Consequently, a qualitative assessment of the improvement in the surface layer condition was performed, taking into account parameters such as surface roughness.

## 2. Materials and Methods

### 2.1. Materials

Six different polymeric materials, unmodified with fillers, produced by Ensinger GmbH (Nufringen, Cham, Rottenburg-Ergenzingen, Germany) and used for the production of products by machining, characterized by a semi-crystalline structure, were used for the study. Tecapet white polyester is characterized by its toughness, good sliding properties, high wear resistance and low coefficient of friction. In addition, it is easy to process and has high chemical resistance. It can be used, for example, in the food industry and in areas where solvents or cleaning agents are present, thanks to the lack of axial porosity. Polyetheretherketone (PEEK)—Tecapeek natural—is characterized by high temperature resistance and high wear and creep resistance. It can be used for the production of elements subjected to heavy loads. Tecaform AH natural acetal is POM-C without fillers, a copolymer used in many industries. It has high stiffness and mechanical strength and excellent wear resistance. In addition, it is characterized by low moisture absorption. It works well in dynamically loaded elements. The Tecaform AD natural material used for the tests is a POM-H acetal homopolymer with good sliding properties and high wear resistance. It is characterized by a higher density and hardness compared to POM-C. Thanks to its good insulating properties, it is easy to process. PA 6 natural is a material with high abrasion resistance and average dimensional stability. Its advantage is its resistance to dynamic loads, maintained also at low temperatures. Due to its good chemical resistance, it is used in harsh environments. Polyamide 66 natural is a wear-resistant material, characterized by good stiffness, hardness, abrasion resistance and thermal dimensional stability. It is used, for example, in the automotive industry, forestry, mining or the electronics industry. Materials manufactured by Ensinger in the form of plates were used in the tests.

### 2.2. Annealing Process

The annealing process was carried out for each of the materials. The annealing process was planned on the basis of preliminary DSC measurements. The parameters and method of heat treatment were selected on the basis of our own research and the literature [12,13,14,15,16]. Heating was carried out in a heat-resistant vessel in oil Polsil OM-10000 (Sip, Nowa Sarzynia, Poland) according to the following parameters: heating rate—0.015 °C/s, heating time—for each 1 mm of thickness, the sample was heated for 900 s, at 0.01 °C/s. The temperature at which the samples were annealed was selected based on the catalog data of the polymer manufacturer and our own research. The selected temperature values were within the range of the highest intensity of crystallization of the tested materials.

### 2.3. Thermal Properties

The tests of the thermal properties of the materials before and after the annealing process were carried out using the DSC differential scanning calorimetry method, based on the measurement of changes in the physical properties of the test substance during a controlled heating process. The DSC 214 Polyma (Netzsch GmbH, Selb, Germany) was used for the tests. The tests were performed in accordance with the PN-EN ISO 11357-3:2018-06 standard [17]. Samples cut from the core, intended for milled boards, were heated at a rate of 10 K/min in the temperature range selected on the basis of the appropriate catalog values for the tested materials. The samples were weighed using a Sartorius scale with accuracy of 0.01 mg, with the possibility of internal calibration and closing the measuring space. The weight of the samples was in the range of 8–12 mg. Measurements were carried out in a nitrogen atmosphere. Graphs of changes in the DSC signal (mW/mg) as a function of temperature were recorded. Data processing and the determination of thermal parameters were carried out in the Netzsch Proteus program. The program makes it possible to study the course of sample melting in a specific temperature range and to determine the surface area between the thermographic curve and the baseline in the range of endothermic reflex. Using the DSC method, the following parameters were determined: the melting temperature range of the crystalline phase, the maximum temperature at which the crystalline phase melts at the highest rate, the melting enthalpy and the degree of crystallinity. The effects of transformations occurring during the melting of polymers were determined from the DSC curves measured for samples heated at a constant rate. The enthalpy of melting of the crystalline phase for a fully crystallized polymer and other parameters were treated as material constants; they can be found in many papers on the measurement of the thermal properties of polymers, as well as in the databases of data analysis programs.

### 2.4. Dynamic Mechanical Properties

The dynamic mechanical properties were determined using the DMA 242 device from Netzsch (Selb, Germany), equipped with a holder for the three-point bending of a beam-shaped sample with dimensions of 55 × 10 × 4 mm. The DMTA tests, performed in accordance with the PN-EN ISO 6721-1:2019-07 standard [18], consisted of causing periodically varying stresses in the samples. For the circular frequency ω = 2πf, the experiment was equivalent to the instantaneous experiments for time t = l/ω [19,20,21,22,23]. By applying a sinusoidally varying stress ε to a sample, a sinusoidally varying strain σ is induced. In addition, it is possible to reveal the angular displacement relative to the stress, which indicates the viscoelastic properties of the material. The test sample was loaded with a sinusoidal force with a frequency of 1 and 10 Hz and heated at a rate of 2 °C/min to the plastic flow temperature determined on the basis of the results obtained from the DSC tests. The conservative modulus E’ and the loss tangent tan δ were recorded. The obtained results are presented in the form of graphs of the above-mentioned quantities depending on the temperature and vibration frequency. Samples of polymeric materials after the annealing process were subjected to analogous tests as before thermal treatment.

### 2.5. Machining of Samples

Samples for DMTA tests were cut from the board core using a Doosan Puma TT1800SY machine lathe equipped with driven tools (Doosan Machine Tools, Seoul, Korea), and data on the milling cutter used are presented in Table 1.

Before starting the processing of the samples, a 3D model of the processed blank was created separately for the material before and after annealing. Based on the created 3D models, a path was generated for the milling cutter. Based on the preliminary tests, the following cutting parameters were selected for machining: the feed rate of the cutter was 600 mm/min; the tool rotated at a constant rotational speed of 8000 rpm. Stocks were machined at a constant cutting depth of 2.5 mm, with a tool engagement width of 40%. The generated program in the form of a single pass of the milling cutter cutting the material with the side surface was uploaded to the machine’s cache. The treatment of both heated and unheated material was carried out on a milling machine in which the material was clamped in a vice. To ensure that the material was placed in the vice, the samples from the bottom were supported at a previously prepared distance, because the height of the samples did not allow for free resting on the bottom of the vice in order to level the sample before processing. This support had an impact on the leveling of vibrations of the workpiece in comparison to fixing the element without support; the lack of vibrations significantly affected the quality of the machined surface.

A comparative analysis of the roughness parameters of the tested materials before and after heat treatment, obtained from the Formtracer SV-C4500 profilometer (Mitutoyo, Kawasaki, Japan), was carried out. The Ra parameter reacts poorly to local changes in surface structure, which is why its value often does not give an objective picture of the surface condition. For this reason, the surface roughness parameter Rz was additionally selected to describe the surface condition.

## 3. Results and Discussion

**Figure 1:**
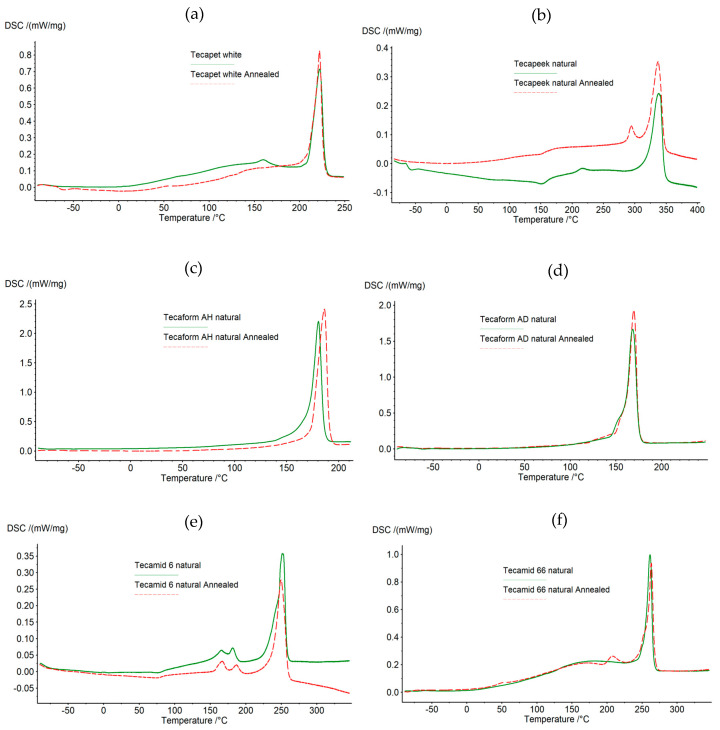
DSC thermograms of the tested materials before and after the annealing process: (**a**) Tecapet white, (**b**) Tecapeek natural, (**c**) Tecaform AH natural, (**d**) Tecaform AD natural, (**e**) Tecamid 6 natural, (**f**) Tecamid 66 natural.

Figure 1a–f show the thermograms obtained for the samples before and after the annealing process, while Table 2 contains a list of the parameters determined on the basis of the DSC measurements.

In the case of testing Tecapet white, it was found that the annealing of the sample affected the thermal properties and the degree of crystallinity of the tested polymer. The degree of crystallinity of the annealed material increased by 2.13% compared to the unannealed polymer. Based on the research, it can be concluded that the melting point range was slightly extended. By analyzing the thermograms for Tecapeek natural, it was found that the annealing of the sample affected the thermal properties and the degree of crystallinity of the tested material. The degree of crystallinity of the annealed polymer increased by 3.88%. An extension of the melting phase range was recorded. By analyzing the thermograms for Tecaform AH, it was found that the annealing of the sample had the desired, positive effect on the thermal properties and the degree of crystallinity of the tested material. The degree of crystallinity of the annealed polymer increased by 4.73%. There was also observed a shift in the maximum reflection of the melting point by 5.9 degrees from the temperature value of 180.8 °C for the unannealed sample to 186.5 °C for the annealed sample. The melting point range shifted towards higher values. In the case of Tecaform AD natural, it was noticed that the degree of crystallinity of the annealed material increased by 5.8% compared to the non-annealed sample. It was also noticed that the melting temperature range did not change significantly, but the amount of energy consumed by the annealed polymer changed for the value of the maximum reflex temperature, which was 169.3 °C for the annealed material and 168.7 °C for the non-annealed material.

In the case of Tecamid 6 natural, the degree of crystallinity was reduced by 0.85%, which proved the slow cooling of the material used for testing in the production process. However, a shift in the beginning of the melting of the crystalline phase towards higher temperature values for the material after the annealing process was registered. By analyzing the thermograms obtained for Tecamid 66 natural, it was found that the annealing of the sample had a positive effect on the thermal properties and the degree of crystallinity of the tested material. The degree of crystallinity of the annealed polymer increased by 7.9%. An increase in the temperature of the end of the melting range of the crystalline phase was observed.

Annealing causes the development of crystal structures. The structure of the investigated polymers after annealing was characterized by an increase in the size of the crystalline structures. The crystallization of the polymer proceeds through the nucleation process, i.e., thermo-dynamically stable nucleation, and through the process of growth of the crystalline phase. Crystals grow much faster in pre-embryos formed, rather than being evenly distributed in the amorphous phase. The formation of any crystal growth process is initiated by the earlier formation of a nucleus with a large surface area in relation to its mass. The degree of chain branching and the molecular weight distribution of the studied polymers significantly affected the crystallinity, which was an important factor affecting the performance properties of the tested polymers—hence the differences in the range of changes after annealing.

In the case of the tested materials, the method of preparation and the thermal history of the samples also affected the mobility of macromolecular segments and the nucleation, growth and orientation of crystallites. The glass transition temperature depends on the chemical and molecular structures of the polymer. For all the tested materials, lower values of the glass transition temperature after the annealing process were recorded, with the largest differences obtained for Tecapet white, Tecapeek natural, Tecamid 6 natural and Tecamid 66 natural. These dependencies were confirmed by the tests carried out using the DMTA method.

**Figure 2:**
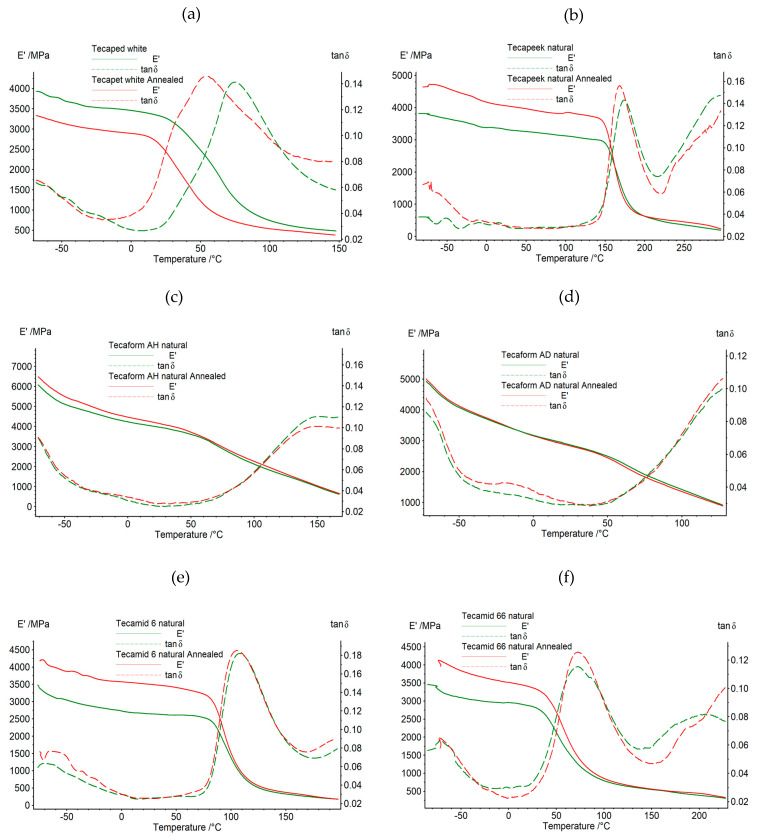
The dependence of the conservative modulus value and mechanical loss tangent versus temperature at a frequency of 1 Hz before and after the heat treatment process of tested materials: (**a**) Tecapet white, (**b**) Tecapeek natural, (**c**) Tecaform AH natural, (**d**) Tecaform AD natural, (**e**) Tecamid 6 natural, (**f**) Tecamid 66 natural.

**Figure 3:**
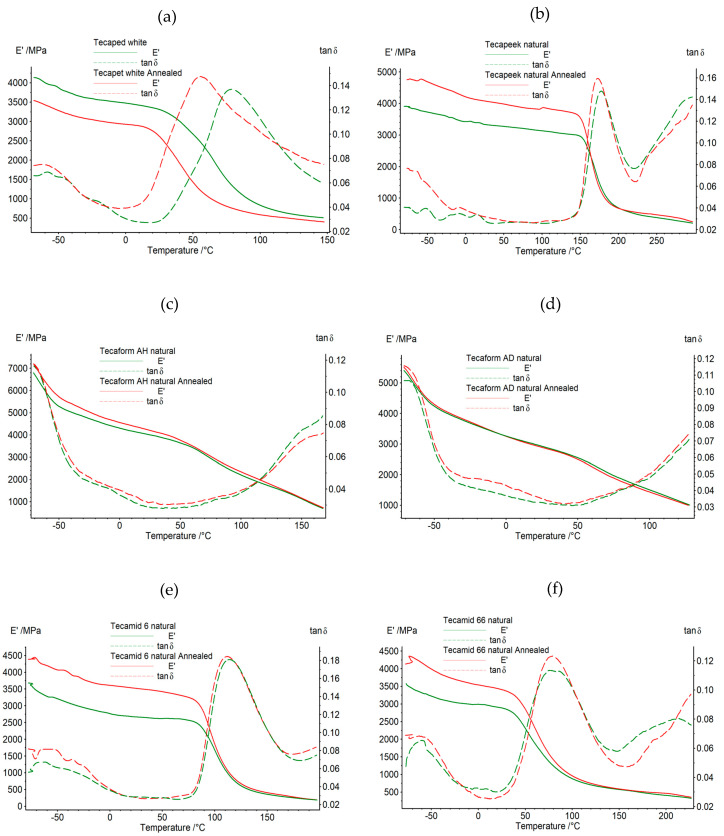
The dependence of the conservative modulus value and mechanical loss tangent versus temperature at a frequency of 10 Hz before and after the heat treatment process of tested materials: (**a**) Tecapet white, (**b**) Tecapeek natural, (**c**) Tecaform AH natural, (**d**) Tecaform AD natural, (**e**) Tecamid 6 natural, (**f**) Tecamid 66 natural.

The results of the tests of changes in the conservative modulus and the tangent of the mechanical loss angle as a function of temperature and vibration frequency for samples before and after the annealing process are shown graphically in Figure 2 and Figure 3. In the case of the tested materials after thermal treatment, significant changes in thermomechanical properties were observed. For the Tecaped white plastic, a similar course of the conservative modulus curve was recorded in the entire temperature range at 1 and 10 Hz excitation. However, differences in the measured values were noted in the entire range of the curve. In the glassy phase, the maximum value of the conservative modulus was 3812 MPa for the material before annealing, while, for the polymer after heat treatment, the maximum value of E’ was 3438 MPa. In the glass transition phase, increasing differences in the value of the conservative modulus were recorded. For the temperature of 50 °C, a decrease in the E’ value of approximately 1238 MPa was recorded for the material after annealing, compared to the material before annealing. As the temperature value increased, smaller differences in the E’ values were recorded. Above the temperature value of approximately 145 °C, i.e., in the final phase of Tecapet’s use, the differences in the values of the conservative modulus were negligible, which means that the material began to flow and did not respond to the excitation force. For the excitation frequency of 1 Hz, before heat treatment, the maximum of the tangent of the mechanical loss angle related to the relaxation transformation was recorded at a temperature of 76 °C, and for the material after heating at 57 °C. In the case of Tecapeek natural, a similar dependence of the tested properties was recorded in the tested temperature range for the excitation frequencies of 1 and 10 Hz.

The greatest differences in the values of the conservative modulus for the material before and after annealing were recorded in the glass phase. As a result of heat treatment, the analyzed samples increased the value of E’ by approximately 18%.

During the further heating of the polymers, no significant differences were observed in the values of the conservative modulus for the samples before and after annealing. Above the temperature value of 300 °C, i.e., in the final range of use, both in the samples before and after annealing, no response to the excitation was recorded, and the value of the conservative modulus approached zero. At the excitation frequency of 1 Hz, for the heat-treated material, the maximum associated with the relaxation transformation was recorded for the temperature value of 172 °C, while, for the unheated material, it was 181 °C. The curves of the conservative modulus and the mechanical loss coefficient for Tecaform AH natural before and after heating were similar in the entire temperature range. For the annealed sample, an increase in the value of the conservative modulus and the tangent of the mechanical loss angle was observed. The largest increase in the value of E’, equal to approximately 410 MPa, was recorded in the glass transition phase. With increasing temperature, smaller differences in the values of the conservative modulus were recorded for the analyzed samples. At temperatures above 145 °C, the values of E’ were very close to each other. In the samples subjected to heat treatment, no significant changes in the value of the glass transition temperature were recorded. In the tan δ diagrams, both before and after annealing, the maximum associated with the relaxation transformation was recorded at the level of approximately −78 °C, for the excitation frequency of 1 Hz. For the Tecaform AD natural material, it was also noticed that in the test temperature range, the materials before and after the annealing process were characterized by similar curves. The analysis of the curves showed that at temperatures lower than −20 °C in the glass transition phase, the value of the conservative modulus was higher for the annealed material by 4 to 7% compared to the unannealed polymer. For the temperature range from −20 to 0 °C, Tecaform AD natural before and after heat treatment had very similar E’ values. With increasing temperature, the unannealed polymer was characterized by higher values, by 20–95 MPa, than the annealed material. Above the temperature value of 130 °C, i.e., in the final range of use, both before and after annealing, the test samples did not show a response to the extortion, and the value of the conservative modulus approached zero. In the case of Tecamid 6 natural, significant changes in thermomechanical properties were recorded after the thermal treatment process. For the conservative modulus, a similar course of the graph was recorded for the entire temperature range of the test at 1 and 10 Hz excitation. However, differences in the measured values were noted throughout the curve. For the excitation frequency of 1 Hz, the maximum value of the conservative modulus of 3492 MPa was recorded in the glass phase for the material before annealing, while, for the polymer after heat treatment, the highest value of E’ was 4178 MPa. In the glassy phase, differences in the values of the conservative modulus between the annealed and unannealed polymer, ranging from 755 to 860 MPa, were observed. A decrease in the difference in E’ values was observed with increasing temperature. For the initial values of the glass transition phase temperature, the discrepancy in the modulus values between the annealed and non-annealed polymer was 576 MPa, while the value of the difference in conservative modulus for the phase end temperature was already 31 MPa at the excitation frequency of 1 Hz. Above the temperature value of approximately 200 °C, i.e., in the final stage of the Tecamid 6 natural polymer’s exploitation, the differences in the conservative modulus values were small, which means that the polymer began to flow and did not respond to the excitation force. For the excitation frequency of 1 Hz, the value of the glass transition temperature was reduced in the annealed material. The maximum value of the mechanical loss coefficient associated with the relaxation transformation was recorded for unannealed Tecamid 6 natural at a temperature of 112 °C, and for the annealed material at 105 °C. Similarly, in the case of Tecamid 66 natural, a similar pattern in the thermographic curves was recorded for the samples before and after annealing in the full temperature range for the excitation frequencies of 1 and 10 Hz. The greatest differences in the values of the conservative modulus for the material before and after annealing were recorded in the glass phase for the temperature value of 60 °C; this difference was 746 MPa at the excitation frequency of 1 Hz, and for the temperature value of the end of the glass phase, 40 °C, the discrepancy in the E’ values between the annealed and non-annealed material was 358 MPa for the excitation frequency of 1 Hz. During the further heating of the samples, in the glass transition phase, the discrepancy in the value of the conservative modulus between the annealed and unannealed Tecamid 66 natural decreased from 283 to 58 MPa at the phase end temperature. During further heating in the phase of highly elastic deformation, no significant differences in the value of E’ were observed. For the excitation frequency of 1 Hz, in the heat-treated material, the maximum tangent of the mechanical loss angle related to the relaxation transformation was 0.12 for the temperature value of 74 °C, while, for the unheated material, it was 0.13 for the temperature value of 71 °C at the excitation frequency of 1 Hz. From the observation of the thermographic curves presenting the results of the mechanical property tests as a function of temperature, determined using the DMTA method, it was noticed that the conservative modulus for almost all tested polymers at the initial test temperature values was increased when comparing the polymer not subjected to heat treatment to the one subjected to heat treatment. On the other hand, in the entire temperature range of the test, E’ showed increased values for the annealed samples compared to the non-annealed ones, for the following materials: Tecapeek natural, Tecaform AH natural, Tecamid 6 natural and Tecamid 66 natural. It was also observed that for the annealed Tecapet white, the shift in the glass transition phase to lower temperature values compared to the non-annealed material was narrowed, while, for Tecamid 66 natural, the glass transition phase when comparing the annealed to the non-annealed polymer was also narrowed.

## 4. Study of the Surface Profile after Machining

**Figure 4:**
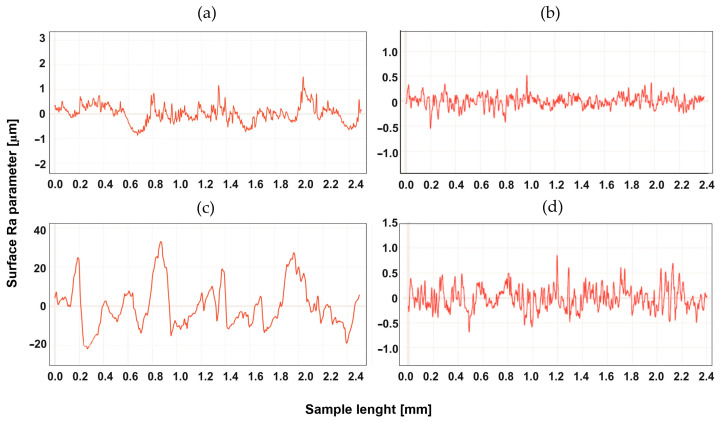
Roughness Ra parameter of Tecapet white, for up-milling (**a**) before annealing and (**b**) after annealing, and for down-milling (**c**) before annealing and (**d**) after annealing.

**Figure 5:**
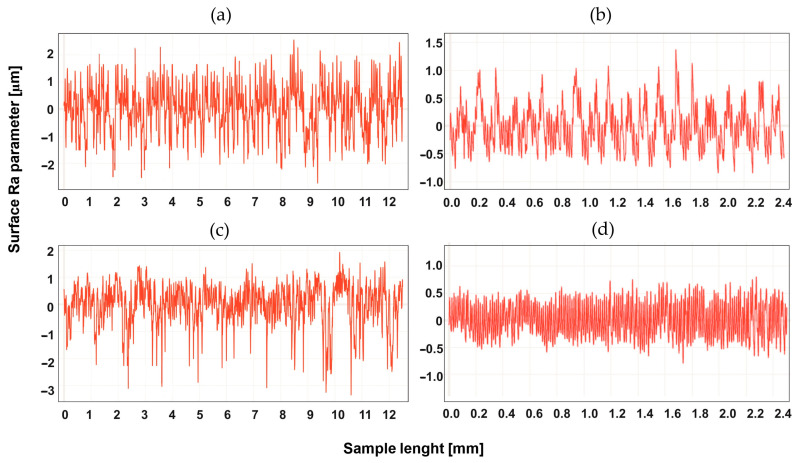
Roughness Ra parameter for Tecapet natural, for up-milling (**a**) before annealing and (**b**) after annealing, and for down-milling (**c**) before annealing and (**d**) after annealing.

**Figure 6:**
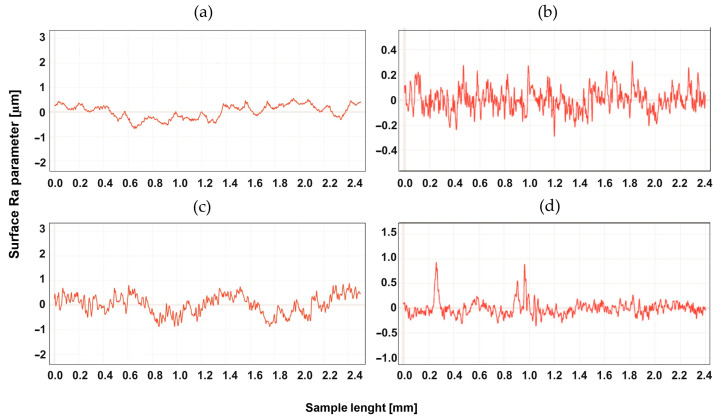
Roughness Ra parameter for Tecaform AH natural, for up-milling (**a**) before annealing and (**b**) after annealing, and for down-milling (**c**) before annealing and (**d**) after annealing.

**Figure 7:**
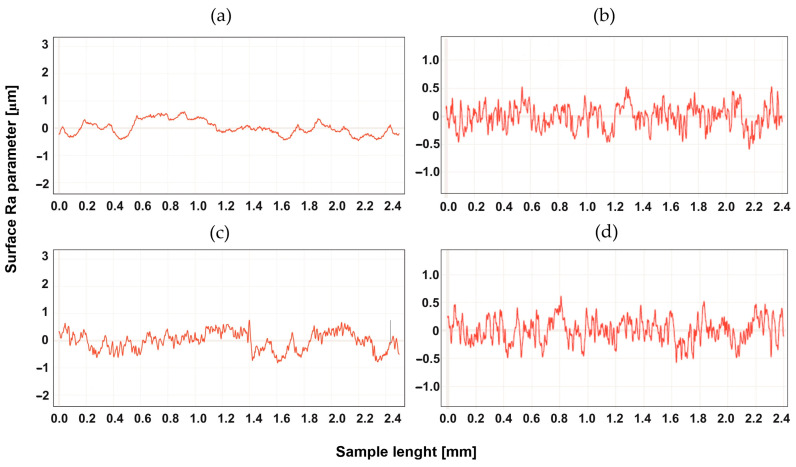
Roughness Ra parameter for Tecaform AD natural, for up-milling (**a**) before annealing and (**b**) after annealing, and for down-milling (**c**) before annealing and (**d**) after annealing.

**Figure 8:**
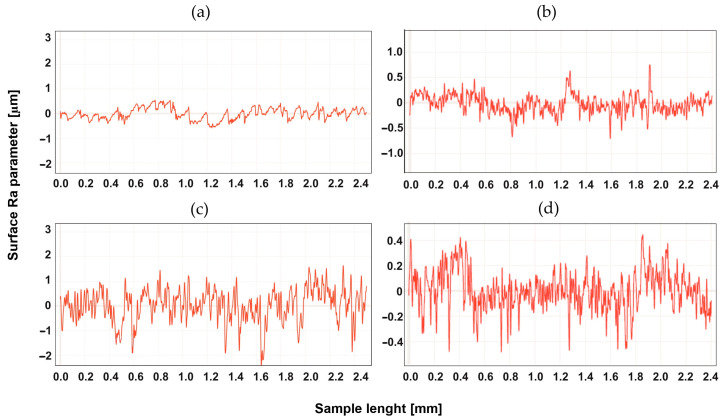
Roughness Ra parameter for Tecamid 6 natural, for up-milling (**a**) before annealing and (**b**) after annealing, and for down-milling (**c**) before annealing and (**d**) after annealing.

**Figure 9:**
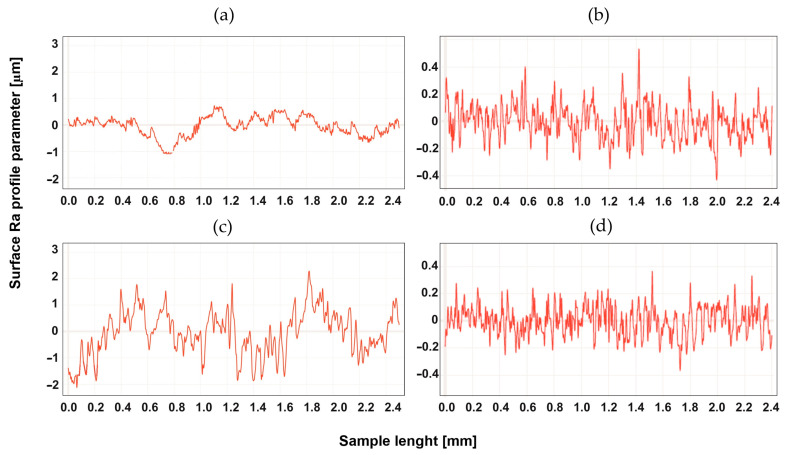
Roughness Ra parameter for Tecamid 66 natural, for up-milling (**a**) before annealing and (**b**) after annealing, and for down-milling (**c**) before annealing and (**d**) after annealing.

The surface development was tested for two types of machining, up-milling and down-milling, for the heated and unheated material. Figure 4, Figure 5, Figure 6, Figure 7, Figure 8 and Figure 9 show the roughness profiles measured for samples before and after annealing.

Significant differences in the roughness profiles were observed for Tecapet white prior to annealing. For down-milling, a profile with a total height 20 times higher than for up-milling was recorded. After the annealing process, the roughness profiles for both types of machining followed similar courses, but it should be noted that the annealing process significantly improved the quality of the surface for down-milling. Figure 5 shows the surface profiles for Tecapeek natural.

The profiles of the tested material before annealing were similar. The annealing process resulted in a significant reduction in roughness for both types of machining in a similar range. It was found, however, that for up-milling, the Tecapeek natural’s surface was shaped in a different way than for down-milling—characteristic faults could be observed, which were not visible in the profile for up-milling. Figure 6 shows the profiles for Tecaform AH natural. In this case, the annealing process did not significantly change the profile. There were also no particular differences between the profiles for up-milling and down-milling. Figure 7 shows the roughness profiles for Tecaform AD natural.

The profiles for Tecaform AD natural, as well as for Tecaform AH natural, were similar. A slight improvement was visible for the annealed material. However, there were no differences in the profiles measured for different methods of machining. Figure 8 shows the roughness profiles for Tecamid 6 natural.

In this case, a clearly different roughness profile for the unheated material could be seen, depending on the type of processing. For down-machining, a surface with a greater degree of surface development was observed. The process of heating the material caused a significant reduction in the roughness profile. The roughness profiles for Tecamid 66 natural are shown in Figure 9.

As for Tecamid 6 natural, the roughness profile for Tecamid 66 natural showed lower values of roughness parameters for climb machining. This difference disappeared after the material was heated.

Data from the analysis of the roughness profiles are presented in Table 3 and Table 4.

For most cases, a positive effect of heat treatment on the surface roughness of the processed materials was observed. When evaluating the roughness parameters (Ra, Rz), it was noticed that the largest average change in the difference between the material heated and not heated (expressed as a percentage) for all materials, regardless of the roughness parameter, was the most favorable for down-milling. The average for the Ra parameter was 25.26% for up-milling and 50.09% for down-milling, while the average for the Rz parameter was as follows: for up-milling 12.37%, for down-milling 48.18%. The plastics that were characterized by the greatest improvement in quality after annealing for the Ra parameter during up-milling were Tecapeek natural, Tecamid 66 natural and Tecamid 6 natural. For down-milling, they were Tecapet white and Tecamid 66 natural. Polymers characterized by the greatest changes after annealing for the Rz parameter after up-milling were Tecapeek natural, Tecamid 66 natural and Tecapet white. For down-milling, the largest changes in the Rz parameter were found for Tecapet white and Tecamid 66 natural.

## 5. Conclusions

1. The thermal modification of polymeric materials has a significant impact on the degree of crystallinity. The analysis of the tests performed showed that the tested polymer materials, with the exception of Tecamid 6 natural, showed a positive effect of the assumed thermal treatment.
2. Heat treatment has a significant impact on the thermal properties of polymeric materials, including temperature ratings. It was found that for most of the tested polymers, the melting temperature range of the crystalline phase changed between the unheated and heat-treated samples, and only one of the materials was characterized by a similar melting range of the crystalline phase—Tecaform AD natural.
3. Properly planned heat treatment makes it possible to improve the machinability of selected polymeric materials by changing their thermomechanical properties, including the strength and stiffness of the material. The curves of the tangent of the mechanical loss angle and the conservative modulus for materials showing the greatest positive effect of heat treatment showed a similar course to the unannealed samples, while, in the entire test temperature range, the values of the conservative modulus were higher for materials after annealing.
4. The conducted process of the heat treatment of polymers by modifying their thermomechanical parameters showed a positive effect on the machinability of the polymer materials, including significant changes in roughness parameters after milling.

## Figures and Tables

**Table 1:** Technical data of the milling cutter used for machining.

Cylindrical Milling Cutter with Polished Flutes	
Nominal diameter	∅ 8 mm
Nominal diameter manufacturing tolerance	e8
Number of blades	5
Helical angle	45°
Blade length	33 mm
Total length	58 mm
Tool holder diameter	∅ 8 mm
Corner chamfer angle	90°
Tool material	VHM

**Table 2:** Thermal parameters determined on the basis of DSC measurements for the tested materials.

Material	Crystallinity Degree [%]	Enthalpy of Melting [J/g]	Range of Melting Points of the Crystalline Phase [°C]	Melting Maximum Reflex Temperature [°C]	Glass Transition Temperature [°C]
Tecapet white	28.51	39.92	210.2–227.8	221.9	81.3
Tecapet white annealed	30.64	42.9	208.7–227.9	221.7	78.1
Tecapeek natural	27.57	35.84	321.8–346.9	338.1	152.1
Tecapeek natural annealed	31.45	40.89	325.6–347.1	336.9	141.7
Tecaform AH natural	79.35	146.8	172.2–185.7	180.4	– 57.4
Tecaform AH natural annealed	84.08	156.4	173.6–190.7	186.2	– 58.6
Tecaform AD natural	35.67	116.3	161.7–175.2	168.3	– 58.2
Tecaform AD natural annealed	41.47	135.2	161.2–174.4	169.4	– 59.3
Tecamid 6 natural	16.05	30.49	245.8–257.4	251.2	78.9
Tecamid 6 natural annealed	15.20	28.89	248.8–259.2	249.4	75.2
Tecamid 66 natural	24.81	48.38	254.1–265.7	261.2	48.2
Tecamid 66 natural annealed	32.71	63.79	258.6–267.8	263.2	44.6

**Table 3:** Surface roughness parameters (Ra and Rz) measured by tactile profilographometer Formtracer SV-4500. P—up-milling, W—down-milling.

Material	Before Annealing				After Annealing			
	Ra [μm]		Rz [μm]		Ra [μm]		Rz [μm]	
	P	W	P	W	P	W	P	W
Tecapet white	0.282	8.683	2.379	54.734	0.179	0.226	1.225	1.752
Tecapeek natural	0.694	0.569	4.646	4.628	0.238	0.166	1.306	1.415
Tecaform AH natural	0.248	0.327	1.232	1.753	0.162	0.192	1.107	1.230
Tecaform AD natural	0.214	0.281	1.067	1.571	0.174	0.175	1.099	1.076
Tecamid 6 natural	0.189	0.515	1.126	4.086	0.106	0.124	1.034	1.121
Tecamid 66 natural	0.287	0.679	1.887	4.405	0.112	0.109	0.865	0.790

**Table 4:** Percentage changes in Ra and Rz parameters of materials after the annealing process.

Material	Ra		Rz	
	P	W	P	W
Tecapet white	36%	97%	48%	97%
Tecapeek natural	66%	71%	72%	69%
Tecaform AH natural	34%	42%	10%	30%
Tecaform AD natural	18%	38%	–3%	31%
Tecamid 6 natural	44%	76%	8%	73%
Tecamid 66 natural	61%	84%	54%	82%

## Data Availability

Data are contained within the article.
